# Stability of the Influenza Virus Hemagglutinin Protein Correlates with Evolutionary Dynamics

**DOI:** 10.1128/mSphereDirect.00554-17

**Published:** 2018-01-03

**Authors:** Eili Y. Klein, Deena Blumenkrantz, Adrian Serohijos, Eugene Shakhnovich, Jeong-Mo Choi, João V. Rodrigues, Brendan D. Smith, Andrew P. Lane, Andrew Feldman, Andrew Pekosz

**Affiliations:** a Department of Emergency Medicine, Johns Hopkins School of Medicine, Baltimore, Maryland, USA; b Center for Disease Dynamics, Economics and Policy, Washington, DC, USA; c Department of Epidemiology, Johns Hopkins University, Bloomberg School of Public Health, Baltimore, Maryland, USA; d W. Harry Feinstone Department of Molecular Microbiology and Immunology, Bloomberg School of Public Health, Johns Hopkins University, Baltimore, Maryland, USA; e Department of Chemistry and Chemical Biology, Harvard University, Cambridge, Massachusetts, USA; f Department of Biochemistry, Cedergreen Center for Bioinformatics and Genomics, Faculty of Medicine, University of Montreal, Montreal, Quebec, Canada; g Department of Otolaryngology-Head and Neck Surgery, Johns Hopkins University School of Medicine, Baltimore, Maryland, USA; Boston University School of Medicine; University of Illinois at Urbana-Champaign; Clemson University

**Keywords:** evolution, hemagglutinin, influenza, protein stability

## Abstract

Protein thermodynamics are an integral determinant of viral fitness and one of the major drivers of protein evolution. Mutations in the influenza A virus (IAV) hemagglutinin (HA) protein can eliminate neutralizing antibody binding to mediate escape from preexisting antiviral immunity. Prior research on the IAV nucleoprotein suggests that protein stability may constrain seasonal IAV evolution; however, the role of stability in shaping the evolutionary dynamics of the HA protein has not been explored. We used the full coding sequence of 9,797 H1N1pdm09 HA sequences and 16,716 human seasonal H3N2 HA sequences to computationally estimate relative changes in the thermal stability of the HA protein between 2009 and 2016. Phylogenetic methods were used to characterize how stability differences impacted the evolutionary dynamics of the virus. We found that pandemic H1N1 IAV strains split into two lineages that had different relative HA protein stabilities and that later variants were descended from the higher-stability lineage. Analysis of the mutations associated with the selective sweep of the higher-stability lineage found that they were characterized by the early appearance of highly stabilizing mutations, the earliest of which was not located in a known antigenic site. Experimental evidence further suggested that H1N1 HA stability may be correlated with *in vitro* virus production and infection. A similar analysis of H3N2 strains found that surviving lineages were also largely descended from viruses predicted to encode more-stable HA proteins. Our results suggest that HA protein stability likely plays a significant role in the persistence of different IAV lineages.

**IMPORTANCE** One of the constraints on fast-evolving viruses, such as influenza virus, is protein stability, or how strongly the folded protein holds together. Despite the importance of this protein property, there has been limited investigation of the impact of the stability of the influenza virus hemagglutinin protein—the primary antibody target of the immune system—on its evolution. Using a combination of computational estimates of stability and experiments, our analysis found that viruses with more-stable hemagglutinin proteins were associated with long-term persistence in the population. There are two potential reasons for the observed persistence. One is that more-stable proteins tolerate destabilizing mutations that less-stable proteins could not, thus increasing opportunities for immune escape. The second is that greater stability increases the fitness of the virus through increased production of infectious particles. Further research on the relative importance of these mechanisms could help inform the annual influenza vaccine composition decision process.

## Abstract (precis)

One of the constraints on fast-evolving viruses, such as influenza virus, is protein stability, or how strongly the folded protein holds together. Despite the importance of this protein property, there has been limited investigation of the impact of the stability of the influenza virus hemagglutinin protein—the primary antibody target of the immune system—on its evolution. Using a combination of computational estimates of stability and experiments, our analysis found that viruses with more-stable hemagglutinin proteins were associated with long-term persistence in the population. There are two potential reasons for the observed persistence. One is that more-stable proteins tolerate destabilizing mutations that less-stable proteins could not, thus increasing opportunities for immune escape. The second is that greater stability increases the fitness of the virus through increased production of infectious particles. Further research on the relative importance of these mechanisms could help inform the annual influenza vaccine composition decision process.

## INTRODUCTION

Seasonal strains of influenza virus are under constant immunological pressure from preexisting population immunity and escape through the accumulation of mutations at important antigenic sites, primarily located in the hemagglutinin (HA) protein (1). Periodically, human influenza A virus (IAV) strains are replaced by strains from animal reservoirs due to the fact that there is little preexisting immunity in the human population, resulting in pandemics (2). While the initial mutations appearing in pandemic influenza viruses likely improve adaptation to the human host (3), they may also come at a thermodynamic cost at the protein level. Thus, protein thermodynamics may constrain seasonal influenza virus evolution (4). Despite its potential importance, the relationship between the thermodynamic stability of the hemagglutinin protein and viral evolutionary dynamics has not been well studied.

Human seasonal IAV evolutionary dynamics are characterized by the survival of only a small number of lineages from year to year, which form the trunk of the evolutionary tree, and many short-lived branches of lineages that come to a dead end (5–7). When novel influenza A virus strains enter the human population, the pH optimum for fusion can change, presumably to improve virus transmission (8). Strain survival in subsequent years of circulation is believed to be primarily due to the presence of adaptive mutations in the surviving lineages compared to the accumulation of other, likely deleterious, substitutions that arise in lineages that die out (7). Often, antigenic changes in the HA protein are thought to drive that adaptive process. Therefore, later variants are the descendants of only a few progenitor viruses (6). Predictions of influenza virus evolution hinge on the fitness differences between these coexisting lineages (7, 9). The difficulty in predicting successful lineages is that survival depends not only on intrinsic fitness differences but also on the multiple stochastic factors that affect successful virus replication, immune evasion, and transmission. Improved understanding of how specific fitness parameters drive evolutionary dynamics could aid in the prediction of IAV antigenic drift variants.

Thermal stability is one of the major biophysical properties that drives protein evolution (4, 10–14). Though the realized fitness of a particular viral strain is a complex phenomenon that is the result of interactions between viral and host molecules, a large fraction of observed mutational effects on fitness parameters (e.g., cell receptor binding or fusion) are also associated with changes in the thermodynamic stability of viral proteins (15). To improve understanding of the relationship between HA protein thermodynamic stability and influenza virus seasonal evolutionary dynamics in the human population, we estimated changes in HA protein thermodynamic stability for both H1N1 and H3N2 strains collected over 7- and 8-year time frames, respectively. Our data demonstrate correlations between HA thermal stability, viral fitness, and continued virus strain circulation in the human population that has important implications for the prediction of global virus circulation and vaccine strain selection.

## RESULTS

### H1N1 phylogenetics and thermodynamic stability.

**FIG 1:**
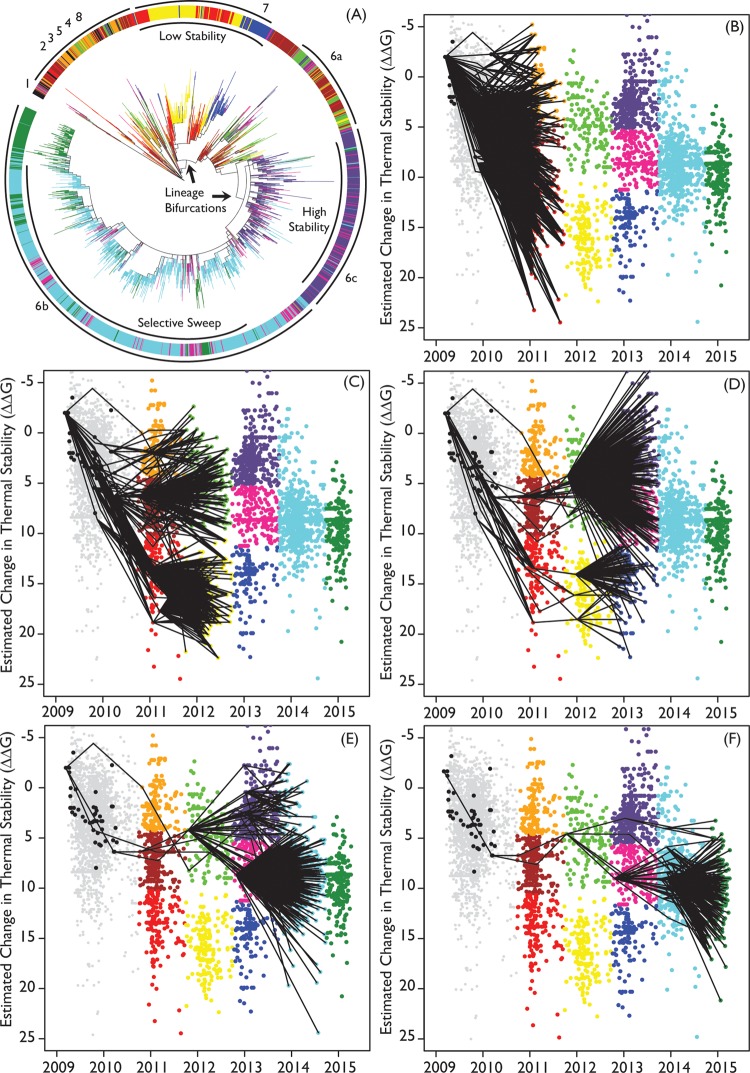
Phylogenetic examination of pandemic H1 HA**.** Each phylogenetic branch and corresponding dot are colored according to their season (1 October to 30 March) and by calculated ΔΔ*G* cluster (see Materials and Methods). (A) Maximum likelihood phylogeny of HA genes of 2,874 nonduplicate pdmH1N1 IAVs. (B to F) Graphs of estimated change in thermal stability of each HA as related to A/California/7/2009 HA over time. Each dot represents a unique HA sequence. Overlaid lines connect most likely ancestors (MLA) of each HA from the specified year back through time. Each line reflects the calculated lowest genetic difference (highest relatedness) between HA sequences from two seasons. Multiple lines were drawn for ties. Once the MLAs for the rightmost sequences were identified, these were the starting sequences for estimating the MLAs in the next prior season. This analysis was continued until the 2009 season for each starting season. In each season, fewer viruses were identified as being more closely related from the prior season, suggesting that there were genetic bottlenecks from one season to the next.

To study the relationship between the thermodynamic stability of the HA protein and viral evolutionary dynamics, we computationally estimated the change in folding free energy (ΔΔ*G*) using the Eris algorithm (16) (see Materials and Methods), compared to the vaccine strain A/California/07/2009 (Global Initiative on Sharing All Influenza Data [GISAID] strain identifier [Id] EPI_ISL_29577), for 9,797 H1N1pdm09 monomer HA sequences that had full open reading frames and date information and were isolated between 12 March 2009 and 9 April 2015. The majority of estimated ΔΔ*G* values were positive (i.e., destabilizing and deleterious), and the estimated ΔΔ*G* of HA declined over the studied period (see Fig. S1 in the supplemental material). Computational estimates were compared to a set of experimental analyses using a thermal denaturation assay (see Materials and Methods), and we found a high degree of correlation (*P* < 0.001) between the estimated ΔΔ*G* and the temperature at which the HA monomers denatured, with A/California/07/2009 being more stable than the other HAs (Fig. S2). Similar to other fast-evolving proteins (17), ΔΔ*G* values of HA mutations were not normally distributed over the whole period (Fig. S3A) or in any individual year (Fig. S3B to F). We used a k-means clustering algorithm (18) to evaluate how ΔΔ*G* values were related by yearly influenza seasons (see Materials and Methods) and found two clear clusters of estimated HA ΔΔ*G* values for H1N1 strains in 2012 and one to three clusters in other years (Fig. 1).

10.1128/mSphereDirect.00554-17.1 FIG S1 Estimated change in thermal stability (ΔΔ*G*) for all H1 HA sequences in a “Gray dots graph” of HA ΔΔ*G* over time. Each dot is an HA sequence for which an estimated change in stability was measured relative to the A/California/07/2009 vaccine strain plotted on the day it was collected. On average, the estimated stability of the virus decreased. The plotted points are the selected viruses that were used in experiments: CA709 (A/California/07/2009) (GISAID strain Id EPI_ISL_29577), GD11 (A/Guangdong/39/2011) (GISAID strain Id EPI_ISL_124917), KY12 (A/Kentucky/08/2012) (GISAID strain Id EPI_ISL_121246), Par13 (A/Parma/52/2013) (GISAID strain Id EPI_ISL_144778), and OK14 (A/Oklahoma/3645/2014) (GISAID strain Id EPI_ISL_159263). Download FIG S1, PDF file, 0.5 MB. Copyright © 2018 Klein et al. 2018 Klein et al. This content is distributed under the terms of the Creative Commons Attribution 4.0 International license.

10.1128/mSphereDirect.00554-17.2 FIG S2 Estimated versus measured stability. Each H1 HA protein was synthetically engineered, and the melting temperature was determined by analyzing thermal denaturation curves. The correlation between melting temperature and the estimated change in thermal stability relative to the vaccine strain (A/California/07/2009) was determined. Download FIG S2, PDF file, 0.4 MB. Copyright © 2018 Klein et al. 2018 Klein et al. This content is distributed under the terms of the Creative Commons Attribution 4.0 International license.

10.1128/mSphereDirect.00554-17.3 FIG S3 Frequency of H1 HA proteins with estimated ΔΔ*G* values. (A) Histogram of estimated change in thermal stability (ΔΔ*G*) for all H1 HA sequences and for the years 2011 to 2015. (B to F) Separate histograms of ΔΔ*G* values for all H1 HAs isolated within each year. Download FIG S3, PDF file, 0.4 MB. Copyright © 2018 Klein et al. 2018 Klein et al. This content is distributed under the terms of the Creative Commons Attribution 4.0 International license.

We included all nonduplicate H1N1 HA sequences obtained on or after 1 October 2010 in calculations of a phylogenetic tree (19–21) with branches colored by the identified stability clusters (Fig. 1A). The majority of HA proteins of the high-stability cluster of 2011 and some of the HA proteins of the high-stability cluster of 2012 belonged to the largest early clade (5). HA proteins of the low-stability clusters from 2011 to 2013 localized to one branch of the tree (clade 7), while high-stability clusters formed the other branch of the tree (clade 6). Further phylogenetic analysis that calculated the most likely ancestor (MLA) for each strain from the prior year, based on pairwise distance measurements (22) (see Materials and Methods), showed that HA proteins started to diverge in 2011 (Fig. 1B) and by 2012 formed two lineages characterized by separate ΔΔ*G* clusters (Fig. 1C). Both low- and high-stability lineages persisted into 2013. However, in 2013, the high-stability lineage represented the majority of isolates, which were primarily descended from the 2012 higher-stability cluster (Fig. 1D). A selective sweep—an extreme reduction in HA genetic diversity—characterized strains isolated in 2014. Nearly all HA proteins in this year were clustered in a single, medium-stability cluster (clade 6B) descending from the medium-stability cluster of 2013 (Fig. 1E). All the HA proteins isolated in 2015 were descended from this cluster, except for a small number of HA proteins which were descendants of the highest-stability lineage (clade 6B; Fig. 1F). The combination of phylogenetic and stability analyses indicates that, by 2011, there had been a bifurcation of the HA protein into two lineages based on stability and, while the lower-stability lineage (clade 7) died out after 2013, the higher-stability lineage (clade 6) persisted. This higher-stability lineage then split (into clades 6A, 6B, and 6C), and later HA proteins were mostly descended from the medium-stability cluster (clade 6B).

### H1N1 HA thermodynamic stability and viral fitness.

**FIG 2:**
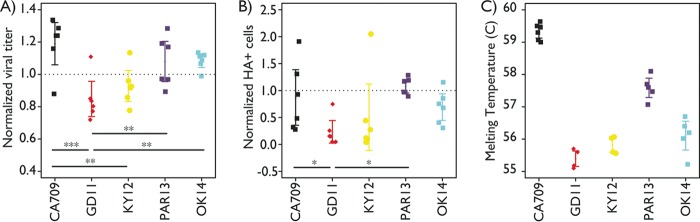
Measures of fitness for recombinant H1N1 viruses. Viruses that encoded different HA proteins on the genetic background of A/California/7/2009 were inoculated into hNEC cultures, and each was normalized to input virus titer and virus with HA from A/California/91/2009 (CA91) to account for hNEC donor variation, and the production of infectious virus particles (A) and the number of infected cells expressing the HA protein (B) were measured 12 h postinfection. The differences in production of infectious virus particles and the number of infected cells expressing the HA protein were related to the measured differences in protein thermodynamic stability (C). The five viral strains are A/California/07/2009 (CA709), A/Guangdong/39/2011 (GD11), A/Kentucky/08/2012 (KY12), A/Parma/52/2013 (PAR13), and A/Oklahoma/3645/2014 (OK14). Statistical significance is indicated by asterisks as follows: *, *P* < 0.05; **, *P* < 0.01; ***, *P* < 0.001.

We assessed the relationship between HA stability and virus replication by infecting primary human nasal epithelial cells with isogenic viruses that differed only in their HA sequences (see Materials and Methods). We infected cells with the same amount of infectious particles of each virus and found that the mean number of infectious particles produced differed significantly between viruses; the virus with the least-stable HA protein generated fewer infectious particles than those with high- and medium-stability HA proteins (*P* < 0.01; Fig. 2A). Similar to virus production, the mean number of epithelial cells infected significantly varied between strains, and the virus with the least-stable HA infected fewer epithelial cells than the two viruses with higher-stability HA proteins (*P* < 0.05; Fig. 2B).

### Amino acid mutations selected during H1N1 HA evolution.

**FIG 3:**
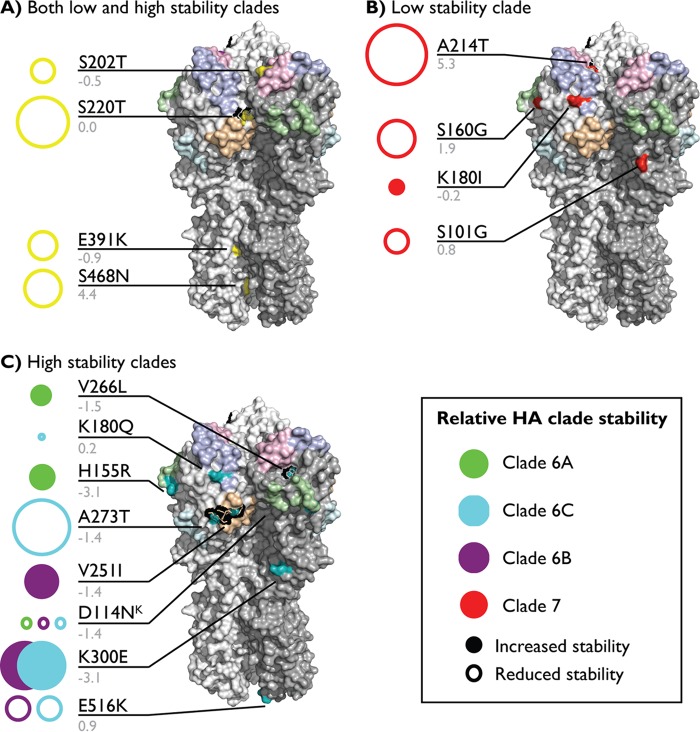
H1 HA amino acid mutations that changed by more than 10% in frequency between 1 year and the preceding year during the studied period. X-ray diffraction crystal structures of the A/California/04/2009 HA trimer (PDB accession no. 3LZG) visualized using MacPyMol v1.8.0.2. (A) Mutations that arose in both clades (yellow). (B) Mutations that arose in the low-stability lineage (clade 7 [red]). (C) Mutations that arose in the high-stability lineage (clade 6A [green], clade 6B [purple], and clade 6C [cyan]). Antigenic sites are colored as follows: Sa (violet), Sb (pink), Ca1 (wheat), Ca2 (green), Cb (cyan). Residue mutation labels are in black, and estimated ΔΔ*G* values (rounded) are in gray. Lines connect residue text with their corresponding amino acids. The areas of the circles are proportional to the estimated ΔΔ*G* values. Closed circles indicate an increase in stability; open circles indicate a decrease.

Analysis of the changes in amino acid frequency of H1 HA proteins identified 19 amino acids that significantly differed over the analyzed time span: 9 amino acids in the high-stability lineage, 5 in the low-stability lineage, and 5 that were in both the high- and low-stability lineages (Fig. S4). In the high-stability lineage, two sets of mutations were associated with rapid increases in frequency suggestive of selection: K300E and V251I, and a triplet mutation consisting of A273T, K180Q, and K300E. The former increased during the 2012-2013 Northern Hemisphere influenza season, and then the V251I mutation declined in frequency as the triplet increased through the 2013 Southern Hemisphere season and became fixed during the 2013-2014 season. Notably, the K300E mutation, which was estimated to increase the stability of the HA protein and was not in a known antigenic site, appeared in collected isolates earlier in 2012 than the other mutations, but did not increase in frequency until the acquisition of the V251I mutation which was located in the known Ca1 antigenic site. In addition, while the V251I mutation was estimated to increase stability, the A273T mutation was estimated to reduce stability (the K180Q mutation was not estimated to impact stability). This contrasts with the mutations in the lower-stability lineage, which were all estimated to reduce stability (Fig. 3).

10.1128/mSphereDirect.00554-17.4 FIG S4 Frequency of individual H1 HA amino acid mutations. Graphs of frequency of prevalence of each of the 19 H1 HA amino acid mutations that rose above 10% penetrance in any year compared to the previous year, grouped by the lineage in which they arose: (A) both lineages, (B) low-stability lineage, (C and D) high-stability lineage. Download FIG S4, PDF file, 0.4 MB. Copyright © 2018 Klein et al. 2018 Klein et al. This content is distributed under the terms of the Creative Commons Attribution 4.0 International license.

### H3N2 phylogenetics and HA thermodynamic stability.

**FIG 4:**
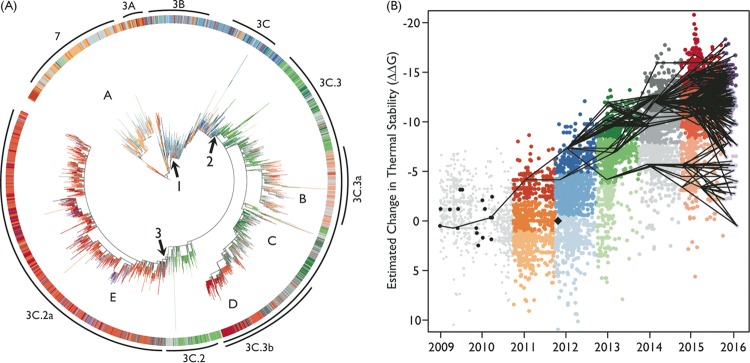
Phylogenetic examination of H3N2 HA. Each phylogenetic branch and corresponding dot were colored according to their season (1 October to 30 March) and ΔΔ*G* cluster. (A) Maximum likelihood phylogeny of HA genes of 8,810 nonduplicate H3N2 IAVs. The three mutational events that introduced the following mutations for the high-stability lineages are indicated by arrows labeled with the numbers 1 to 3. Mutational event 1 introduced A214S, V239I, and N328S. Mutational event 2 introduced Q49R, S61N, T64I, N161S, and N294K. Mutational event 3 introduced N160S, F175Y, K176T, N241D, and Q327H. HA sequence lineages A and B were the identified lower-stability lineages. Lineage A went extinct in 2013 (see Fig. S5 in the supplemental material), while lineage B is linked to the lower line in panel B. Lineages C and D tracked with the higher-stability lineages labeled E but lacked the mutations in group 3 and did not appear to persist into 2016. Multiple small branches are associated with the higher-stability lineage E. (B) Graph of estimated change in thermal stability of each HA as related to A/Victoria/361/2011 (black diamond) over time. Each dot represents a unique HA sequence. Overlaid lines connect most likely ancestors (MLAs) of each HA from the specified year back through time. Each line reflects the calculated lowest genetic difference (highest relatedness) between HA sequences from two seasons. Multiple lines were drawn for ties. Once the MLAs for the rightmost sequences were identified, these were the starting point for the next MLA estimation. This was continued until the 2009 season.

To examine whether changes in HA protein stability were also associated with the evolutionary dynamics of H3N2, we conducted a similar analysis of ΔΔ*G* in 16,716 H3N2 strains relative to the A/Victoria/361/2011 vaccine strain (GISAID strain Id EPI_ISL_101506) collected between 1 January 2009 and 29 January 2016. Unlike H1N1, the average estimated stability of the HA protein in these strains increased over the study period. The k-means clustering algorithm found three stability clusters for each year. Within the time frame analyzed, there were two low-stability H3 HA branches, one that emerged in 2010 and died out after 2013 (clade 7), and another that emerged in 2013 and persisted into 2016, albeit with diminished frequency (clade 3C.3a). The majority of isolates detected in 2015 and 2016 appeared to descend from medium- or high-stability clusters of the preceding year (Fig. 4 and Fig. S5). In general, similar to H1N1, lower-stability H3 HA lineages appeared to be localized to short-lived branches, while lineages of higher stability persisted.

10.1128/mSphereDirect.00554-17.5 FIG S5 H3 HA MLA graphs by year. (A to D) Graphs of estimated change in thermal stability (ΔΔ*G*) of each HA as related to A/Victoria/361/2011 HA (black diamond) over time. Each dot represents a unique HA sequence. Each dot is colored according to the season (1 October to 30 March) and ΔΔ*G* cluster. Overlaid lines connect most likely ancestors (MLAs) of each HA from the specified year back through time. Each line reflects the calculated lowest genetic difference (highest relatedness) between HA sequences from two seasons. Multiple lines were drawn for ties. Once the MLAs for the rightmost sequences were identified, these were the starting point for the next MLA estimation. This was continued until the 2009 season. Download FIG S5, PDF file, 1.7 MB. Copyright © 2018 Klein et al. 2018 Klein et al. This content is distributed under the terms of the Creative Commons Attribution 4.0 International license.

Analysis of the changes in amino acid frequency found 13 mutations that became fixed in the H3N2 viral population and 11 mutations that transiently increased in frequency at least 10% from 1 year to the next but did not become fixed. The 13 mutations that became fixed were in the higher-stability lineages and appeared during three separate selection events (Fig. S6). The first selected group (A214S, V239I, and N328S) initially appeared in October 2010 and swept through the population by the beginning of 2012 and were designated clade 3B. The second selected group (Q49R, S61N, T64I, N161S, and N294K) first appeared in January 2012, and all mutations were present in the majority of isolates by 2013 (clade 3C.2 and clade 3C.3). Finally, we observed the appearance of N160S, F175Y, K176T, N241D, and Q327H in October 2013, which came to dominate collected isolates in 2015 and 2016. All HA clade 3C.2a isolates had this third set of mutations. Notably, though the overall stability of the H3 protein appeared to decrease between 2009 and 2010, in contrast to H1 HA, the majority of H3 HA mutations that were selected between 2010 and 2016 were estimated to be stability enhancing.

10.1128/mSphereDirect.00554-17.6 FIG S6 Frequency of mutations associated with H3 HA selective sweeps. Frequency of prevalence of H3 HA amino acid mutations for the mutational groups that became fixed in the population (A), for each of the 13 amino acid mutations that became fixed in the population grouped by the time in which they arose (B to D), and for each of the transient mutations associated with lower-stability lineages (E and F). Download FIG S6, PDF file, 0.4 MB. Copyright © 2018 Klein et al. 2018 Klein et al. This content is distributed under the terms of the Creative Commons Attribution 4.0 International license.

**FIG 5:**
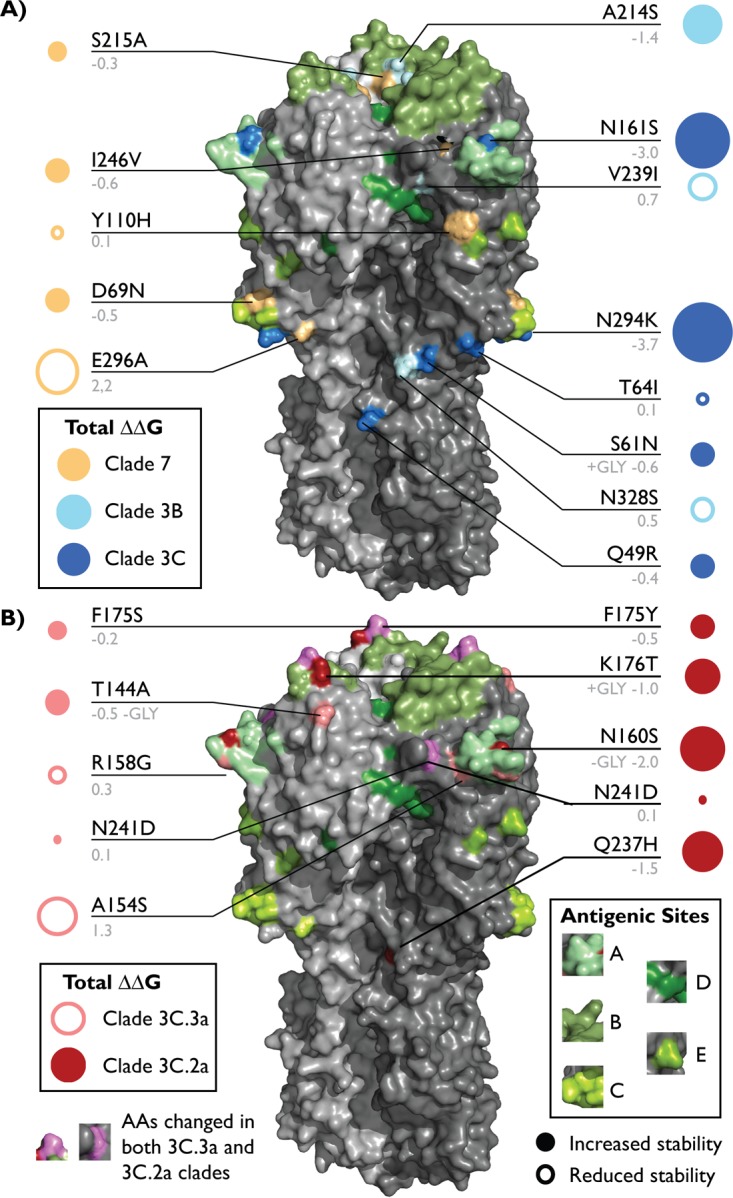
H3 HA amino acid mutations that were selected in indicated clades. X-ray diffraction crystal structures of the A/Aichi/02/1968 HA trimer (PDB accession no. 2YPG) visualized using MacPyMol v1.8.0.2. (A) Mutations that characterized clade 7 (light orange), clade 3B (pale cyan), and clade 3C (marine) are indicated. (B) Mutations that characterize clade 3C.3a (salmon), 3C.2a (brick), or both (violet) are indicated. Antigenic sites are colored shades of green as follows: A (pale), B (smudge), C (limon), D (forest), E (split pea). Lines connect residue text with their corresponding amino acids. Residue mutations are shown in black, and estimated ΔΔ*G* values are in gray. Mutations that altered a putative glycosylation site are noted in red if gained and blue if lost. The areas of the circles are proportional to the estimated ΔΔ*G* values. Closed circles indicate an increase in stability, and open circles indicate a decrease in stability.

Similar to H1N1, comparison of the stability of H3 HA mutations by clade found that lineage longevity was associated with mutations that increased the expected stability of the HA proteins. The primary mutations differentiating clades 3B and 3C from clade 7 were estimated to increase relative stability (Fig. 5A), and those of clade 3C.2a were estimated to be more stable than those of clade 3C.3a (Fig. 5B). The estimated effects of the mutations that differentiated clade 3C.2a from clade 3C.3b were also higher (Fig. S7).

10.1128/mSphereDirect.00554-17.7 FIG S7 H3 HA amino acid mutations that changed by more than 10% in frequency between 1 year and the preceding year. X-ray diffraction crystal structures of the A/Aichi/02/1968 HA trimer (PDB accession no. 2YPG) visualized using MacPyMol v1.8.0.2. (A) Mutations that characterized clade 3C.3b (purple) and 3C.2a (brick) are indicated. Note that V363M is grayed out, because although it was initially selected in 3C.3b, it was later selected against. Antigenic sites are colored shades of green as follows: A (pale), B (smudge), C (limon), D (forest), and E (split pea). Lines connect residue text with their corresponding amino acids. Residue mutations are shown in black, and estimated ΔΔ*G* values are in gray. Mutations that altered a putative glycosylation site are noted in red if a putative glycosylation site was gained and blue if a putative glycosylation site was lost. The areas of the circles are proportional to estimated ΔΔ*G* values. Closed circles indicate an increase in stability, and open circles indicate a decrease in stability. Download FIG S7, TIF file, 1.7 MB. Copyright © 2018 Klein et al. 2018 Klein et al. This content is distributed under the terms of the Creative Commons Attribution 4.0 International license.

## DISCUSSION

We found that the estimated changes in the thermal stability of IAV HA proteins were associated with the evolutionary dynamics of both the H1N1 and H3N2 viral strains. For H1N1, we analyzed the evolution of the pandemic strain after its introduction in 2009. Prior analyses observed a bifurcation in the H1N1 HA phylogeny in which two lineages persisted between 2011 and 2014 before one went extinct (23). Our analysis found that the bifurcation of the viral lineages was characterized by differences in HA protein stability. Furthermore, the surviving lineage was shown to be derived from strains possessing HA proteins of higher estimated thermal stability. An analysis of H3N2 over several seasonal epidemics similarly found that relatively higher stability was associated with long-term lineage persistence.

In our model of HA protein stability, there are two potential reasons for the noted relationship between thermal stability and lineage persistence. (i) HA proteins with higher stability may be relatively more fit regardless of immunological pressure. (ii) Increased stability improves tolerance to acquisition of destabilizing mutations, which in turn allows more opportunities for functionally beneficial or antigenically distinct mutations to be selected. The overall HA monomer stability is a parameter that is inherently different from the pH stability of HA—a factor that is known to be important for the initial adaption of influenza A viruses to new hosts, efficient virus entry into cells, and transmission between mammalian hosts (24).

Experimental evidence from isogenic viruses in which only the HA proteins differed suggests that there may be some fitness advantages to being more stable. Increased production of infectious particles by viruses with more-stable HA proteins may be due to protein properties such as relatively higher folding efficiency (25, 26), stability against degradation in cells (27–29), and resistance to aggregation (30). Lower viral yield could be due to lower infectivity by viruses with less-stable HA proteins. The potential fitness benefit of stability is not the driving force in evolution of the HA protein; however, it likely plays an important role in shaping lineage persistence by reducing transmission of less-stable viruses. This would be expected because the effects of stability on fitness are greatest for HA proteins that are less stable to begin with, as a greater share of mutations are likely to lead to denaturation (12). Above a certain level of stability, the fitness effects are probably lessened or absent (12, 14, 15), which is why we observed a decrease in the overall stability of H1N1 viruses and circulation of viruses of differing stabilities at the same time.

We examined the other alternative of increased tolerance through an analysis of the changes in frequency of amino acid mutations over the study period and found that large selective sweeps in both H1N1 and H3N2 lineages were accompanied by mutations that increased the relative stability of the virus. For example, in H1N1, the stabilizing K300E mutation appeared in the population early in 2012 and increased in frequency rapidly in both 2013 and 2014. Unlike many other mutations that were selected (Fig. 5), K300E, was not located in an antigenic site, nor was it at the HA monomer interface. It is thus possible that the increased stability conferred by K300E was a driver of evolutionary dynamics by allowing strains with this mutation to explore more antigenic drift mutations, including A273T, which was destabilizing, and K180Q, which was coacquired, thus possibly allowing escape at two antigenic sites.

Our H3N2 analysis found three major selection events. However, unlike H1N1, the overall stability of the H3 protein was estimated to increase over the study period, and the most stabilizing mutations were located in antigenic sites. The overall estimated increase in stability of the H3 protein was surprising in light of the H1 analysis; however, while the H1N1 results are, in part, likely related to adaptive changes of the virus to a human host, the changes in stability of H3N2 are the result of regular seasonal changes in epidemiology. Additional research is needed to elucidate the role of stability and selection in this seasonal context. However, it is possible that stability varies due to relative differences in stability-related fitness, with increased stability related to greater fitness. This increase in stability may allow for greater exploration of genetic space, which, in turn, leads to selection of antigenically distinct strains that have relatively lower stability (as most mutations are likely to reduce stability).

There are some limitations to our analysis. Results are based on collected and sequenced isolates uploaded to public databases. Though this encompasses more than 20,000 isolates between the two strains, the distribution both geographically and over time is not constant; thus, there may be some bias in when and where isolate collection occurred. Stability and phylogenetics were based on the sequences deposited in GISAID. This is typically the consensus sequence, and thus ignores other variants that may be present at low concentration that may have evolutionary importance. While prior computational estimates of protein stability for large, complex, multimeric proteins, have provided good estimates of stability (16); past estimates were based on single amino acid substitutions (31). Our estimates of changes in thermodynamic stability were for multiple mutations, and though we assumed that changes in stability were additive, and thus did not account for epistasis, the results of a sample of experimentally measured variants were highly correlated with computational estimates. To our knowledge, this is the first study to show the efficacy of computational stability measurements for multiple mutations in complex proteins. As experimental protein stability measurements are expensive and time-consuming, making their large-scale use impractical, this study demonstrates the power of computation for estimating how stability impacts evolution in future analyses. Last, we did not examine the potential impact of mutations and thermal stability in the other viral proteins that make up the influenza virus.

### Conclusion.

IAV strains are a significant threat to public health causing thousands of deaths annually in the United States (32). The most-effective tool is vaccination; however, rapid evolution of the virus (33) and limitations of the manufacturing process (34) can result in vaccines that are poorly matched for the prevailing seasonal strain (35). Critical to the vaccine composition decision process are predictions of strain evolution in order to assess both which virus is likely to produce the next common variant and the likelihood that the evolved strain will be antigenically distinct from the vaccine strain. Notably, the vaccine strains for H3N2 have been selected from groups estimated to have had relatively lower stability (Fig. S8), which may account for their low efficacy (36). Combined analysis of protein thermal stability and phylogenetics has shown some promise in predicting changes in stability (31). Our results provide evidence that HA protein stability either plays a vital role in determining fitness or is a proxy for fitness-determining factors, and may aid in predicting the emergence of new antigenic variants.

10.1128/mSphereDirect.00554-17.8 FIG S8 Vaccine strain timing for H3N2. Each vaccine strain for H3N2, when it was selected and when it was utilized is shown on the figure. Each strain is listed with a large diamond: A/Perth/16/2009 (orange), A/Victoria/361/2011 (black), A/Texas/50/2012 (red), A/Switzerland/9715293/2013 (blue), and A/Hong Kong/4801/2014 (green). The timing of when each vaccine was utilized is shown with the corresponding colors along the *x* axis. Download FIG S8, PDF file, 0.7 MB. Copyright © 2018 Klein et al. 2018 Klein et al. This content is distributed under the terms of the Creative Commons Attribution 4.0 International license.

## MATERIALS AND METHODS

### Influenza virus strains.

Nucleotide sequences for the influenza virus hemagglutinin (HA) coding region were obtained from the Global Initiative on Sharing All Influenza Data (GISAID) EpiFlu database (platform.gisaid.org). Full-length protein-coding sequences were selected for all A/H1N1pdm09 samples collected from humans from 12 March 2009 (the first recorded A/H1N1pdm09 virus in the database) through 9 April 2015 and for all A/H3N2 samples collected from humans between 1 January 2009 and 29 January 2016. A total of 12,631 H1N1pdm09 and 22,821 H3N2 HA sequences were identified. Of these, 1,603 H1N1 and 6,105 H3N2 HA sequences were excluded because they did not contain the full coding sequence and another 1,222 H1N1 and 192 H3N2 HA sequences were excluded because they lacked a collection date (either month/day/year). Multiple-sequence alignment was done using MAFFT (37, 38) with the FFT-NS-2 progressive alignment algorithm. Multiple-sequence alignment was viewed with ClustalX (39). The HA proteins from five H1N1 viruses, including the vaccine strain, were selected for measurement of protein thermal stability. HA proteins from selected viruses were chosen to span the time period of the study and to include multiple geographic areas and a spectrum of computationally estimated stability levels. The viruses selected were A/California/07/2009 (GISAID strain Id: EPI_ISL_29577), A/Guangdong/39/2011 (EPI_ISL_124917), A/Kentucky/08/2012 (EPI_ISL_121246), A/Parma/52/2013 (EPI_ISL_144778), and A/Oklahoma/3645/2014 (EPI_ISL_159263).

### H1N1 HA protein purification.

HA sequences from ATG through nucleotide 1596, which encodes the last amino acid in the crystal structure were codon optimized, synthesized with a 6×His tag added to the end of each monomer, cloned, and expressed using the customized protein service in the baculovirus expression system (Genescript, Piscataway, NJ). Briefly, the gene was subcloned into a transfer vector. Insect cells were transfected with the recombinant bacmid DNA. Protein quality control was performed by SDS-PAGE and Western blotting (WB) and matrix-assisted laser desorption ionization–time of flight mass spectrometry (MALDI-TOF MS) detection. Proteins were further analyzed by size exclusion chromatography to confirm that the majority of proteins were expressed as monomers in solution.

### Thermal stability measurements.

Purified HA protein was used in thermal stability measurements to gauge the accuracy of computational estimates. Thermal denaturation curves were created by monitoring changes in intrinsic tryptophan fluorescence intensity (excitation at 280 nm and emission at 350 nm) upon protein unfolding using a step temperature increase of 1°C/min. The melting temperature (*T*_*m*_) was determined from the analysis of the thermal denaturation curves with proteins at concentrations of 0.15 mg/ml (*n* = 2 or *n* = 3) and 0.03 mg/ml (*n* = 2 or *n* = 3) by the method of Cimmperman and Matulis (40). Correlation between thermal stability measurements and computational stability estimates was done by linear regression using R (41).

### Generation of recombinant influenza viruses.

Recombinant viruses encoding the selected H1N1 HA proteins were generated. Seven of the eight viral gene segments of each virus had the exact sequence as A/California/7/2009. The HA genes differed only by nonsynonymous nucleotide changes present in their published consensus sequences. In this way, only the effects of mutations in the HA protein would impact viral fitness. All HA genes were synthesized by Blue Heron Biotech (Bothell, WA). The other seven genes of the viruses were cloned from A/California/7/2009 H1N1 virus (kindly donated by Kanta Subbarrao, NIH), and the sequence was confirmed to match the consensus of sequences published in GISAID and GenBank. Each gene segment was cloned into a pHH21 vector, which has a human Pol I promoter and terminator. Recombinant influenza A viruses were generated using a 12-plasmid reverse genetic system as described previously (42–44). An additional sequence was used to standardize infection and replication assays: A/California/VRDL91/2009 (GISAID EPI_ISL_75126). Viruses were recovered from transfected 293T cells (provided by Robert A. Lamb, Northwestern University) followed by coculture with Madin-Darby canine kidney (MDCK) cells (provided by Robert A. Lamb, Northwestern University), plaque purification and amplification on MDCK cells (45). The virus titers were determined by 50% tissue culture infective dose (TCID_50_) on MDCK cells. The entire HA coding region was sequenced to confirm the presence of the desired mutations. The 293T and MDCK cells were obtained by Andrew Pekosz from Robert A. Lamb, Northwestern University, in 1997. The cells were expanded, and stocks of frozen cells were created which are used to seed new cultures to this day.

### Computational thermal stability predictions.

The Eris algorithm (16) was used to predict the relative thermal stability for every complete HA sequence obtained from the GISAID database compared to the vaccine strain A/California/07/2009 for H1N1 and A/Victoria/361/2011 for H3N2. The Eris algorithm calculates the change in the folding free energy (ΔΔ*G* = Δ*G*_mutant_ − Δ*G*_wild type_ where Δ*G*_mutant_ is the Δ*G* of the mutant) using atomistic modeling and a scoring function that accounts for van der Waals forces, solvation, hydrogen bonding, and backbone-dependent energies from amino acid substitutions (46, 47). The folding stability effects of mutations that accumulated on the H1N1 vaccine strain A/California/07/2009 HA sequence (AGM53850.1) and the H3N2 vaccine strain A/Victoria/361/2011 were estimated by introducing them into an HA monomer based on the structure of the strain (PDB accession no. 3LZG and PDB accession no. 4WE8, respectively). We focused on the stability of the monomer, as evidence suggests HA trimer formation occurs spontaneously (without the assistance of intracellular chaperones) through random collisions and oligomerization of HA monomers in solution after transcription and monomer folding in the cell endoplasmic reticulum (48, 49). Thus, correct folding of the monomer is needed for trimer formation, and HA trimer density in the cell and on the surface of the virus would be related to monomer stability. The effects of multiple mutations on folding stability were calculated in an additive manner. We assumed additivity in the effect of multiple mutations, because while epistasis is important, the effects of multiple mutations of unrelated residues are often equal to their incremental binding energy changes (50).

### Virus replication and infectivity.

Cultures of human primary, differentiated nasal epithelial cells (hNECs) were obtained from nondiseased tissue samples from volunteers, as previously described (51, 52), and infected with each of the six influenza viruses on three separate occasions at an approximate multiplicity of infection (MOI) of 0.5 TCID_50_/cell. Infectious virus titers were determined from apical washes, by TCID_50_ as described above. At 12 h postinfection, Accumax (Innovative Cell Technologies) was added to cells, and they were incubated at 37°C for 30 min to create single-cell suspensions. Flow cytometry (FACSCalibur; BD Biosciences) was used to detect the number of infected cells using an anti-influenza virus H1 HA A/California/04/2009 (H1N1)pdm09, monoclonal antibody clone 4F8 (BEI), and Alexa Fluor 488 (AF488)-conjugated secondary donkey anti-mouse (Molecular Probes) antibodies. Differences between strains were calculated using analysis of variance (ANOVA).

### Phylogenetic analyses.

All nonduplicate virus sequences obtained on or after 1 October 2010 were included in calculations of the phylogenetic tree using RAxML Blackbox (19) with the CAT measure of rate heterogeneity and maximum likelihood to estimate the best tree. For viruses with the same nucleotide sequence, the sequence with the earliest date of collection was kept. Additionally, a set of sequence variants from 2009 to 2010 (including the H1N1pdm vaccine strain, A/California/7/2009) were used to ensure rootedness of the phylogenetic tree. The best tree was then projected using the Interactive Tree Of Life (20, 21). A k-means clustering algorithm (18) was used to determine the number of clusters for the computationally estimated ΔΔ*G* values for sequences in each year (1 October to 30 September). A cutoff of 80% for within-cluster sum of squares by cluster was used to define cluster numbers.

We examined how HA sequences from adjacent years were related by calculating the most likely ancestor(s) (MLA) in the prior year for every HA sequence of a specified year (e.g., for an HA sequence with a collection date between October 2012 and March 2013, its MLA was identified as the most closely related HA sequence from all sequences collected between October 2011 and March 2012). MLAs were calculated as the smallest pairwise distance measurements (i.e., highest relatedness) between two strains using the K80 model (22). In the case of ties, both ancestral strains were included. Once MLAs for a season had been calculated, the exercise was repeated using the identified viruses and finding their MLAs in the season prior to the one in which they were identified. This was repeated until the 2009 season was reached. Calculations of pairwise distance were done using the APE package in R (53, 54).

### Frequency of amino acids.

Changes in the frequency of mutations were calculated for all amino acids that were used to calculate changes in thermodynamic stability. For H1N1, HA changes in the frequency of mutations were calculated for yearly periods (1 October to 30 September) from 2011 to 2015. For H3N2, HA changes in the frequency of mutations were calculated for yearly periods (1 October to 30 September) from 2010 to 2016. Changes were considered significant if an amino acid frequency changed by greater than 10% from the preceding year. Amino acids were considered fixed in the population if they were present at a frequency above 70% for more than 2 years.

### Ethics statement.

Human nasal epithelial cells were obtained from nondiseased hosts during endoscopic sinus surgery for noninfection-related conditions. The research protocol was approved by the Johns Hopkins Institutional Review Board. All donors provided written consent, and samples were deidentified and unlinked from all clinical, demographic, and personal data—with the exception of age and sex—before use.

10.1128/mSphereDirect.00554-17.9 TABLE S1 GISAID acknowledgment table for global influenza virus sequences. Download TABLE S1, XLSX file, 1.2 MB. Copyright © 2018 Klein et al. 2018 Klein et al. This content is distributed under the terms of the Creative Commons Attribution 4.0 International license.
