# Supplementary figures and images for: Stability of the Influenza Virus Hemagglutinin Protein Correlates with Evolutionary Dynamics
Source: mSphere. 2018 Jan 3;3(1):e00554-17. doi: 10.1128/mSphereDirect.00554-17 (PMC5750392; doi:10.1128/mSphereDirect.00554-17)

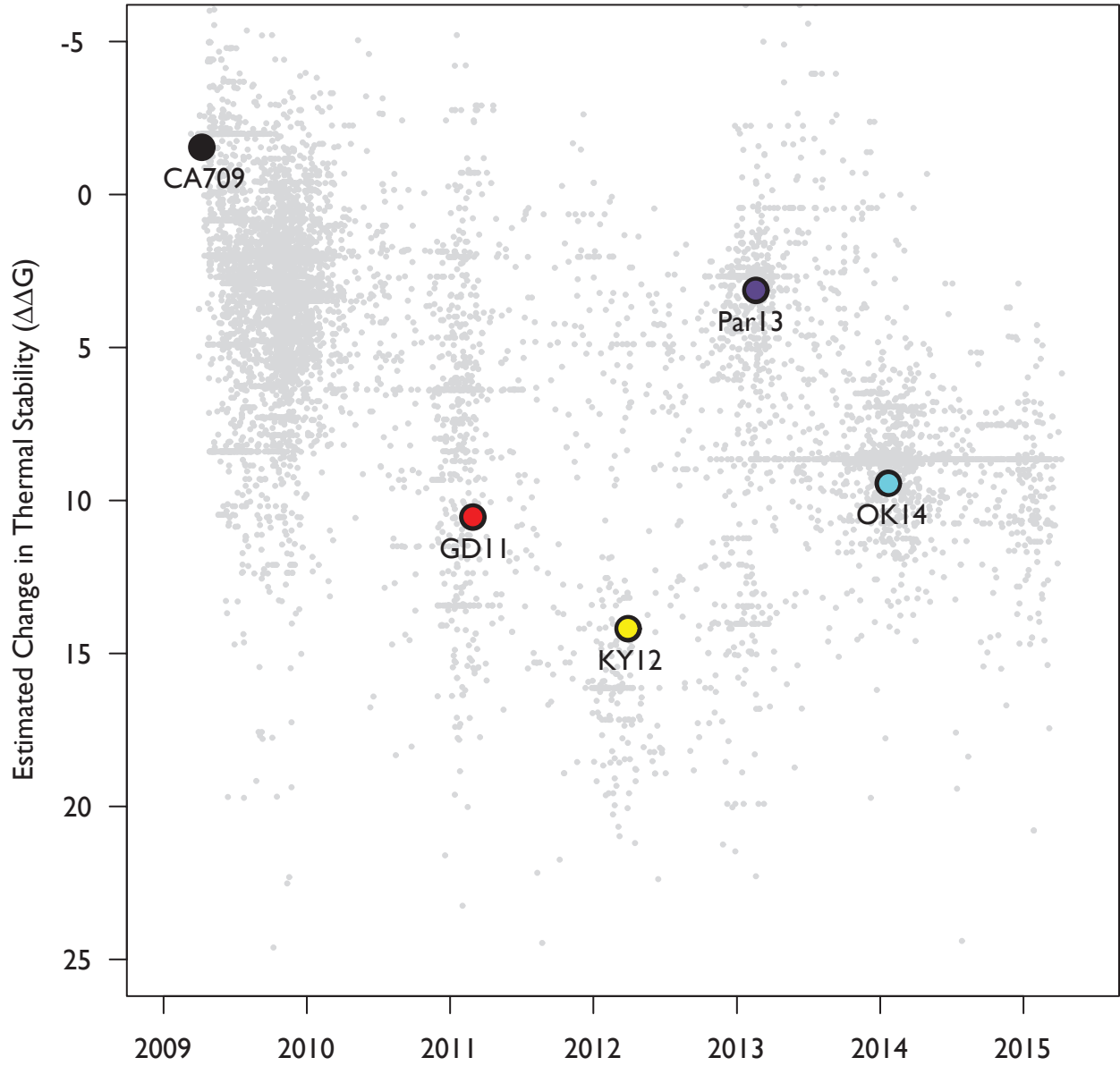

Supplement: FIG S1 [file sph001182442sf1.pdf]

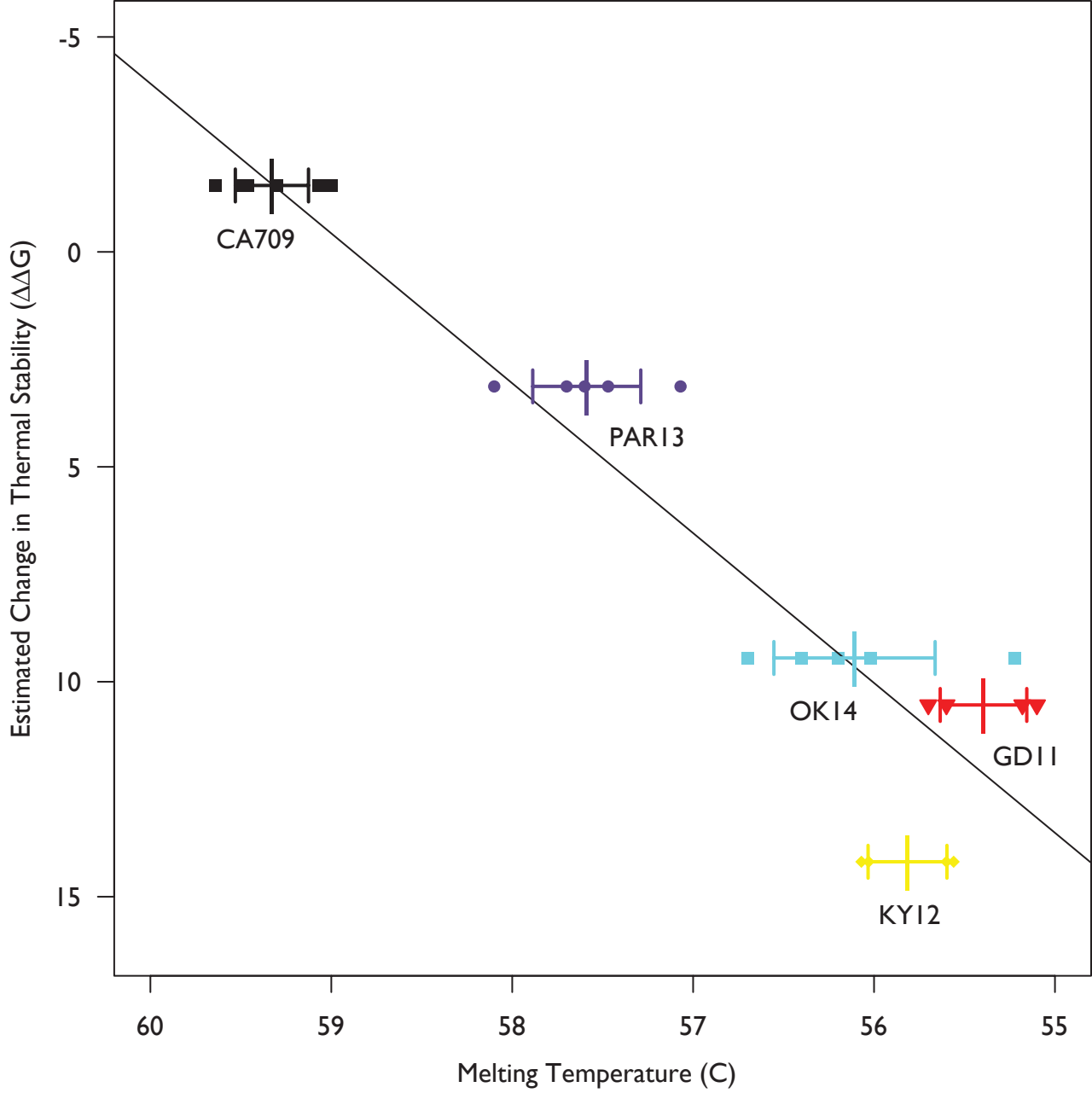

Supplement: FIG S2 [file sph001182442sf2.pdf]

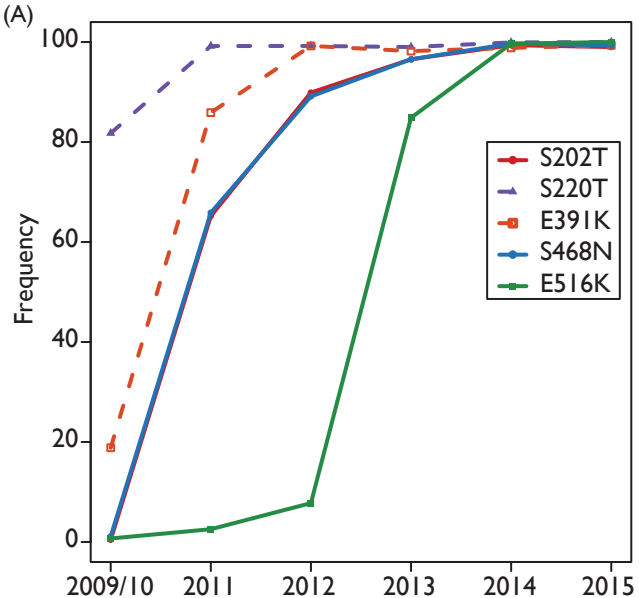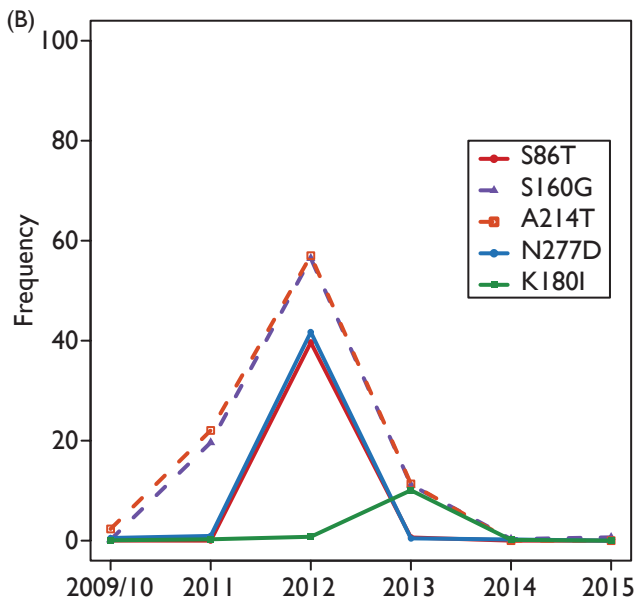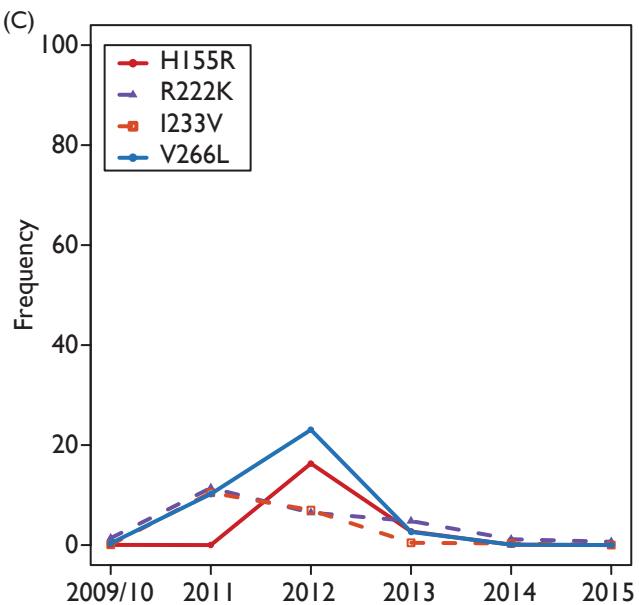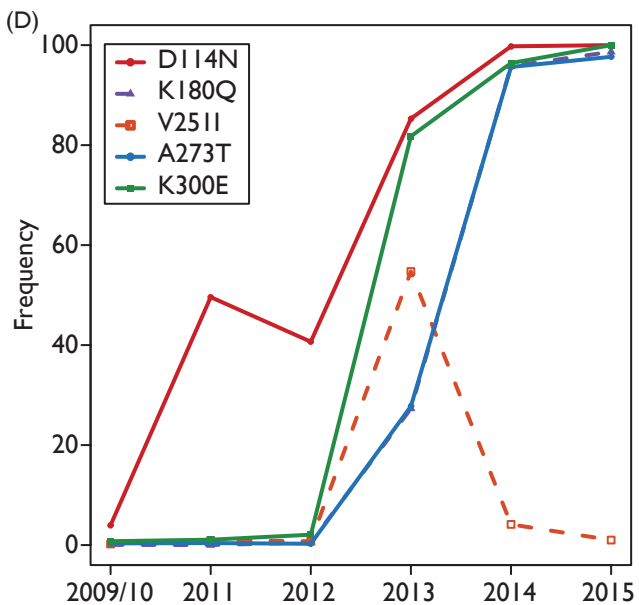

Supplement: FIG S4 [file sph001182442sf4.pdf]

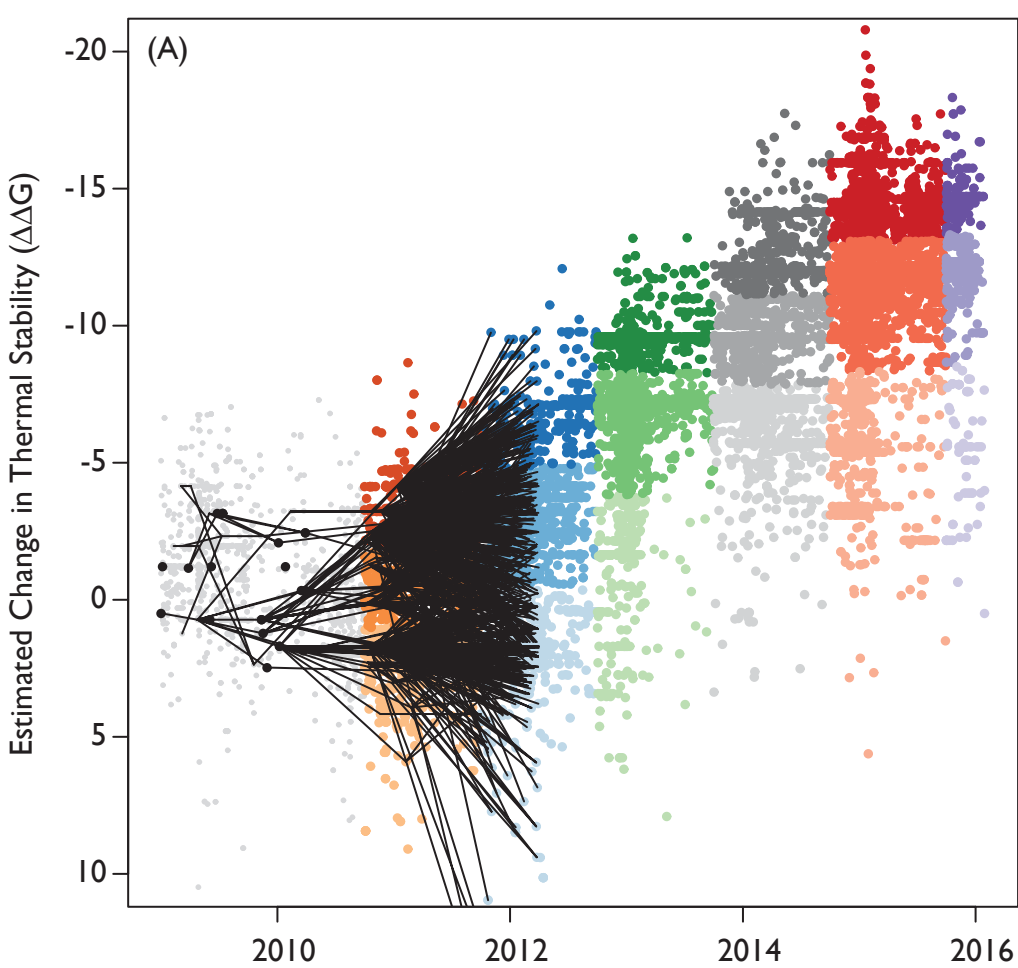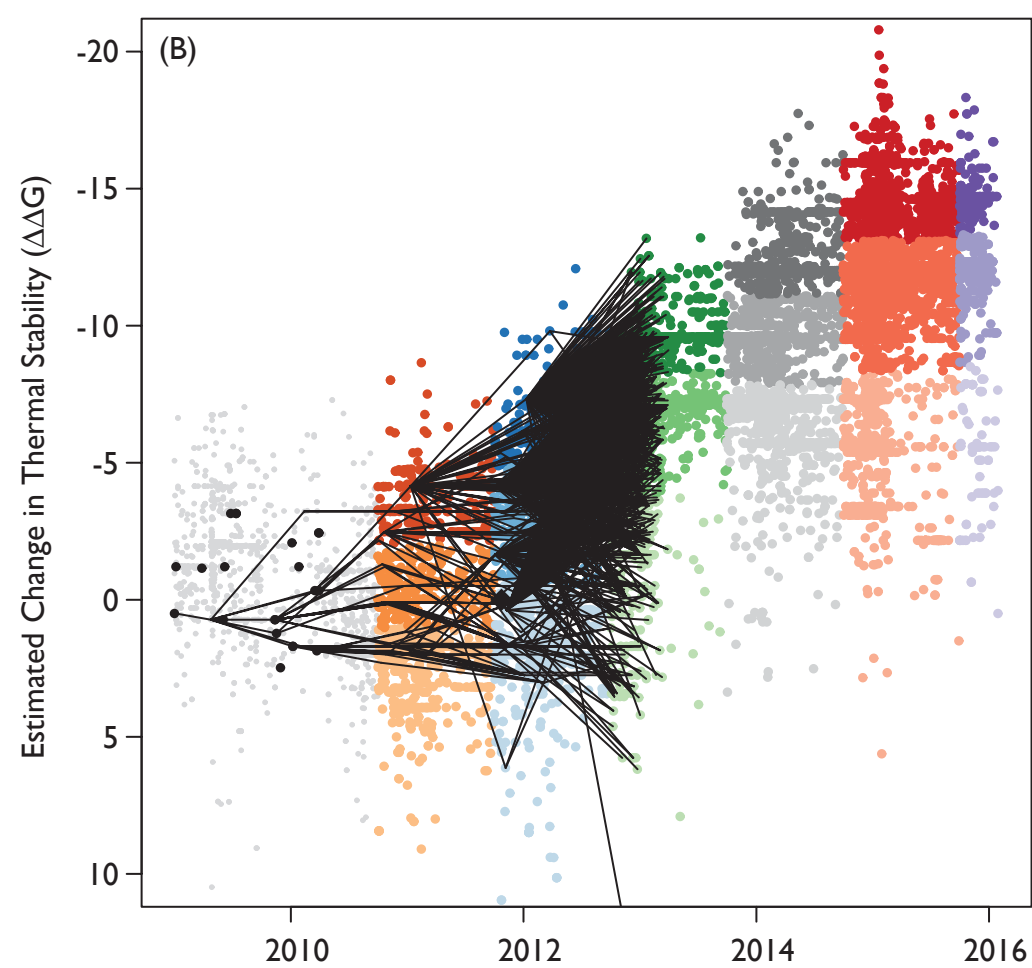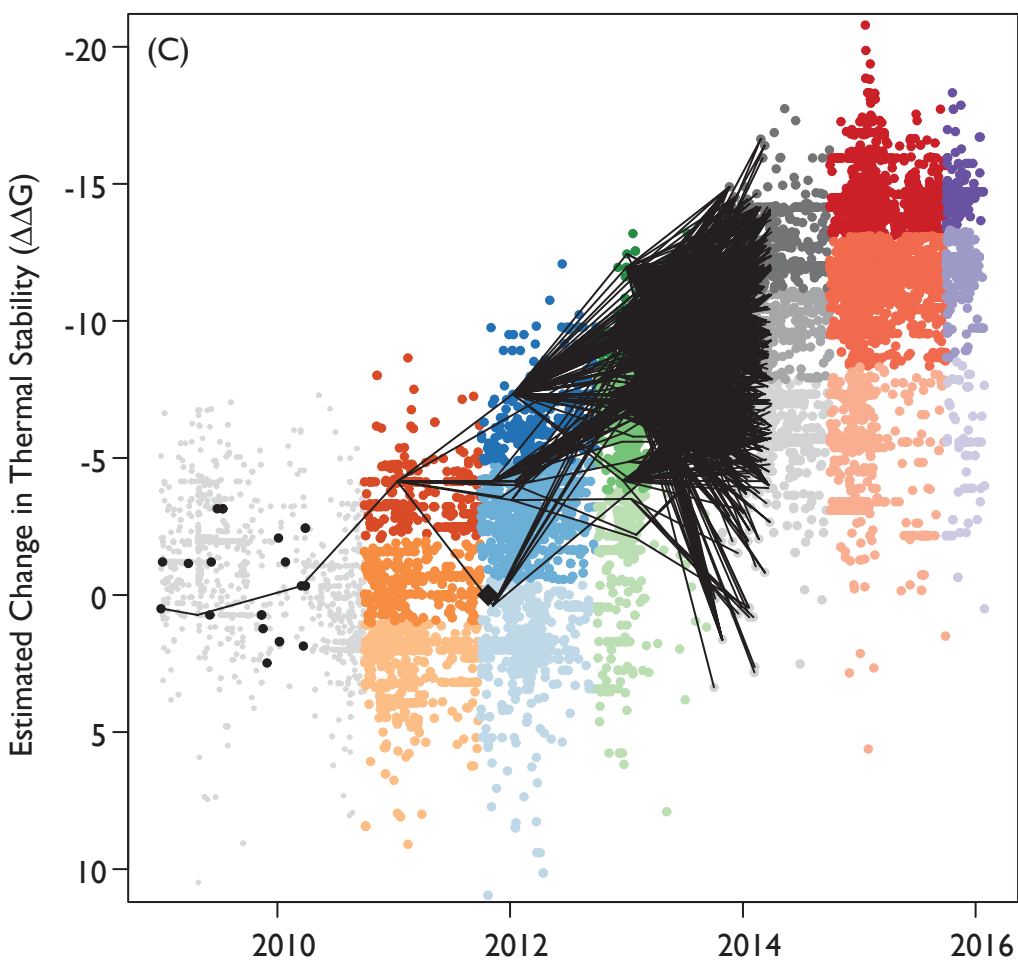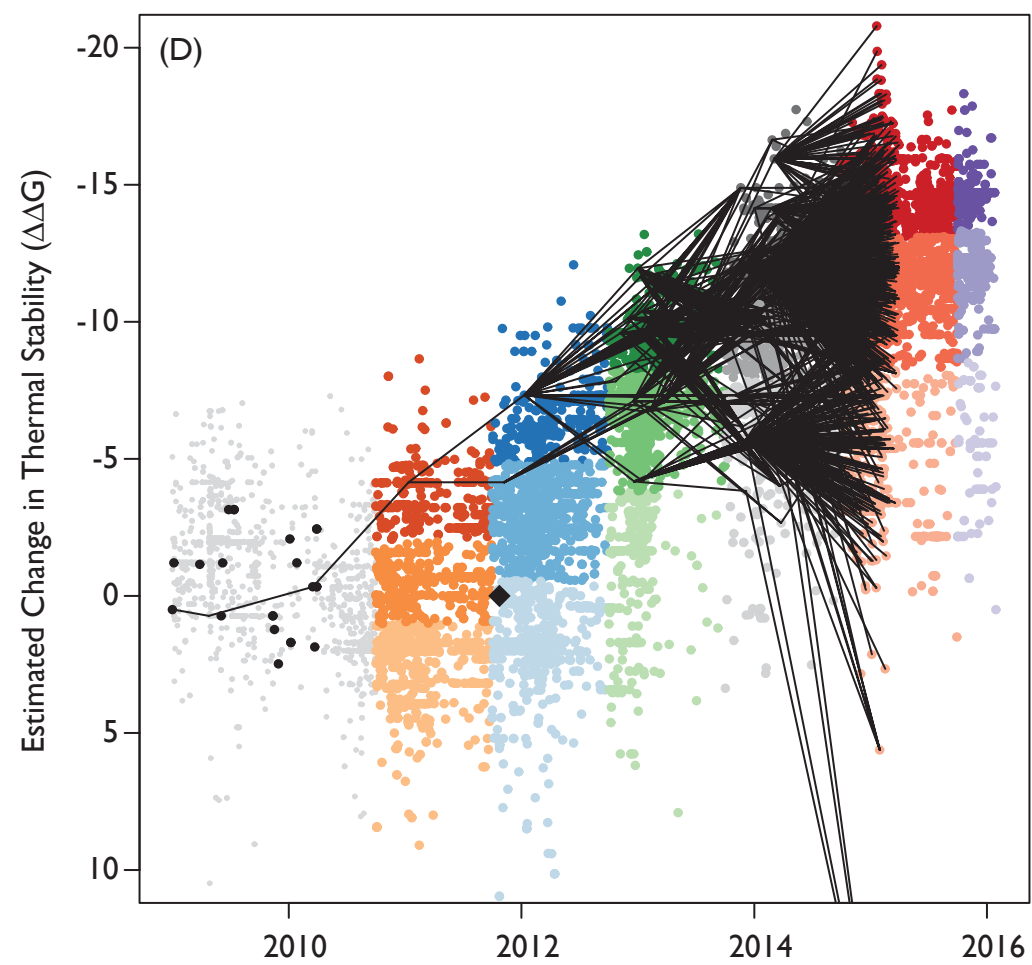

Supplement: FIG S5 [file sph001182442sf5.pdf]

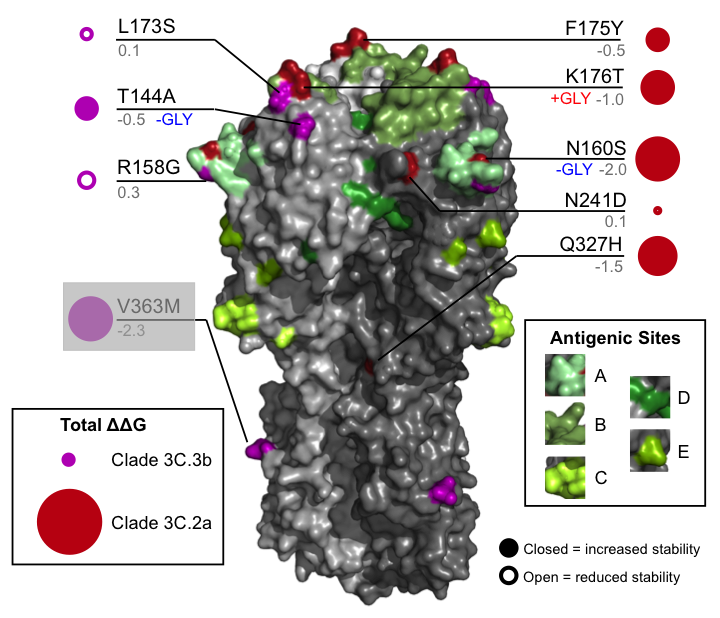

Supplement: FIG S7 [file sph001182442sf7.tif]

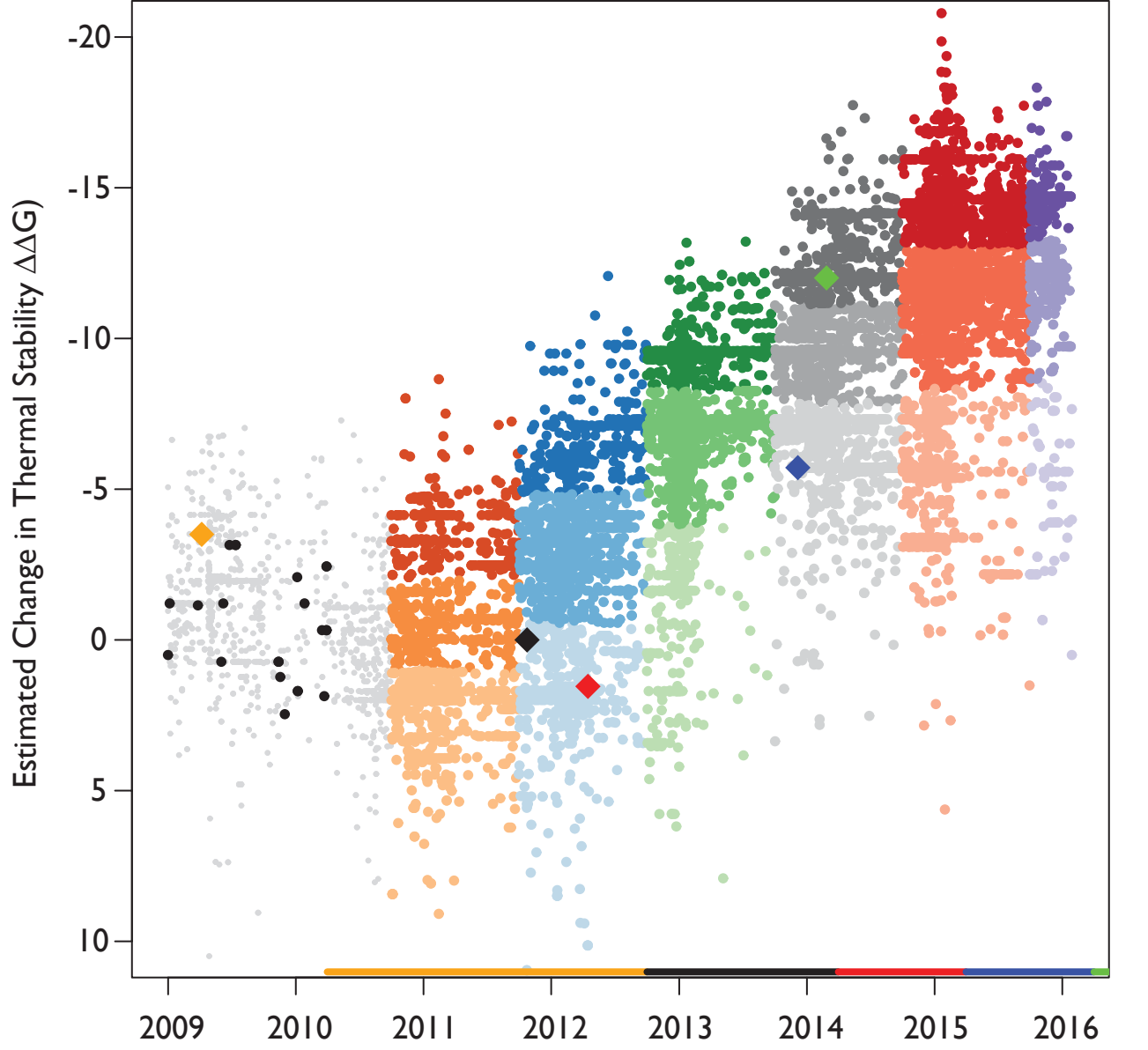

Supplement: FIG S8 [file sph001182442sf8.pdf]
