# Supplementary material for: Stability of the Influenza Virus Hemagglutinin Protein Correlates with Evolutionary Dynamics
Source: mSphere. 2018 Jan 3;3(1):e00554-17. doi: 10.1128/mSphereDirect.00554-17 (PMC5750392; doi:10.1128/mSphereDirect.00554-17)
Supplement: FIG S3 [file sph001182442sf3.pdf]

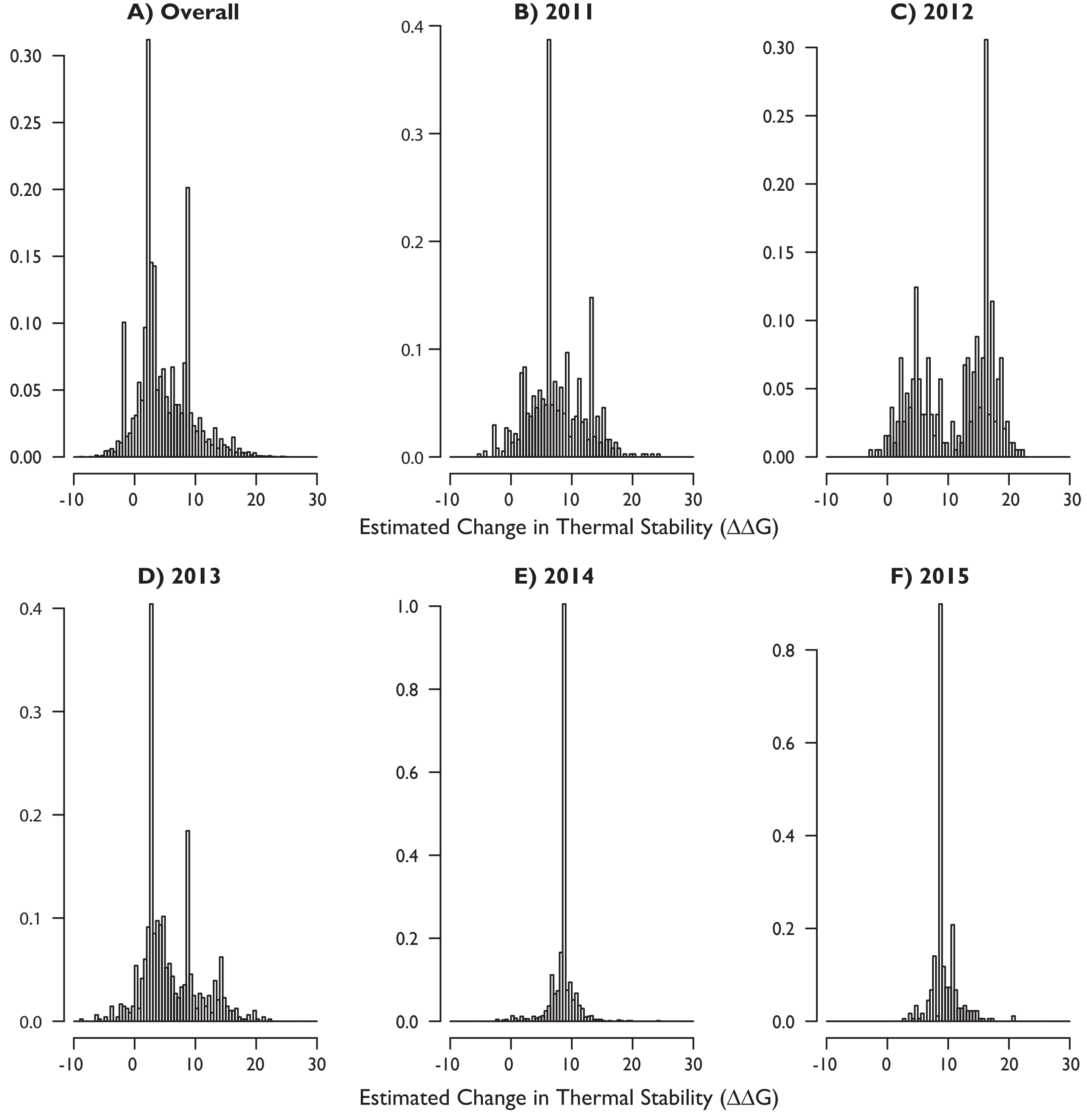
A) Overall
B) 2011
C) 2012
D) 2013
E) 2014
F) 2015
Estimated Change in Thermal Stability (ΔΔG)
Estimated Change in Thermal Stability (ΔΔG)
