# Supplementary material for: Stability of the Influenza Virus Hemagglutinin Protein Correlates with Evolutionary Dynamics
Source: mSphere. 2018 Jan 3;3(1):e00554-17. doi: 10.1128/mSphereDirect.00554-17 (PMC5750392; doi:10.1128/mSphereDirect.00554-17)
Supplement: FIG S6 [file sph001182442sf6.pdf]

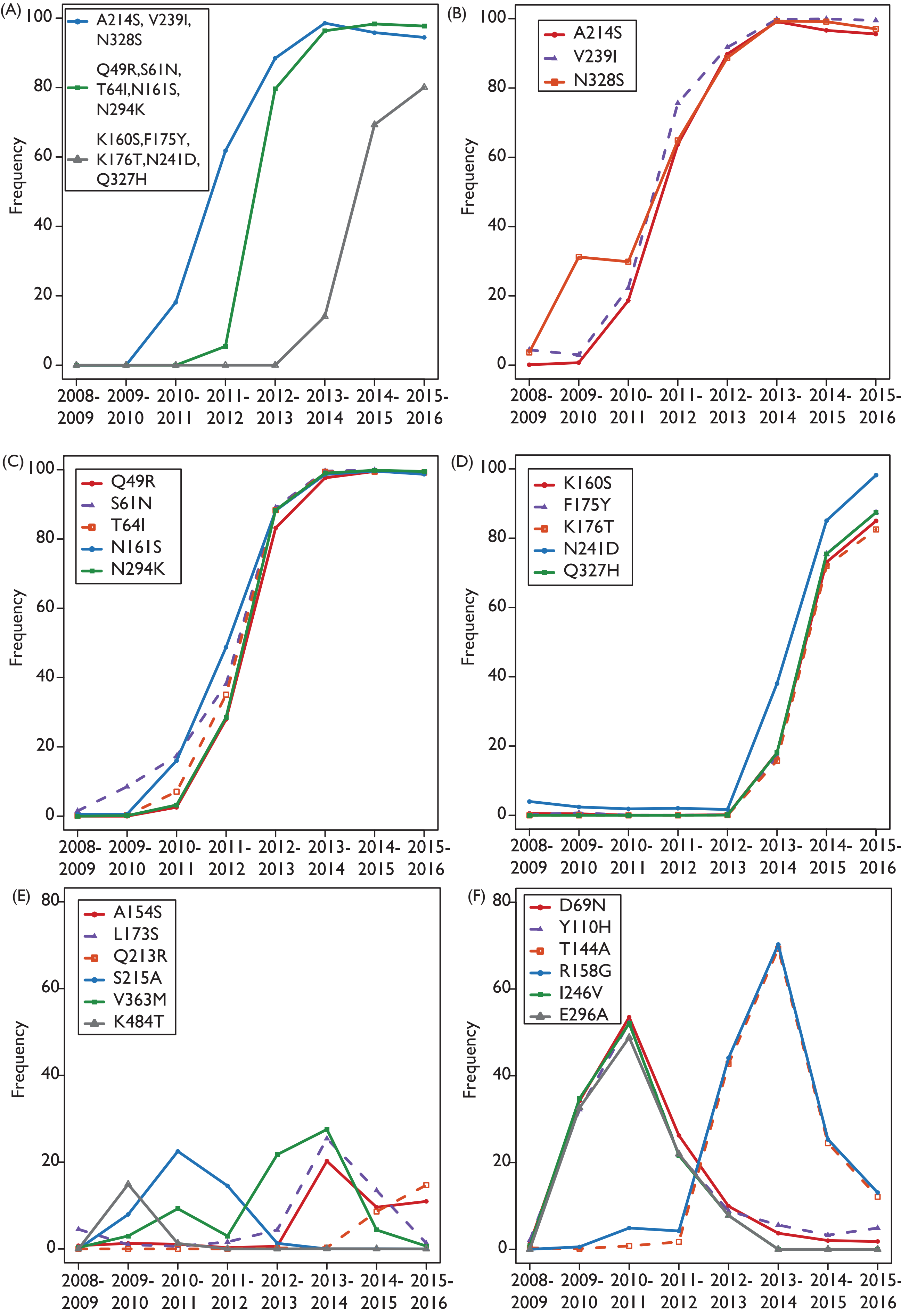
(A)
A214S, V239I, N328S
Q49R,S61N, T64I,N161S, N294K
K160S,F175Y, K176T,N241D, Q327H
(B)
A214S
V239I
N328S
(C)
Q49R
S61N
T64I
N161S
N294K
(D)
K160S
F175Y
K176T
N241D
Q327H
(E)
A154S
L173S
Q213R
S215A
V363M
K484T
(F)
D69N
Y110H
T144A
R158G
I246V
E296A
Frequency
2008-2009
2009-2010
2010-2011
2011-2012
2012-2013
2013-2014
2014-2015
2015-2016
